# EPIGENETIC AGING IN OLDER BREAST CANCER SURVIVORS: THE THINKING AND LIVING WITH CANCER (TLC) STUDY

**DOI:** 10.1093/geroni/igac059.854

**Published:** 2022-12-20

**Authors:** Kelly Rentscher, Traci Bethea, Wanting Zhai, Harvey Cohen, Brent Small, Tim Ahles, Jeanne Mandelblatt, Judith Carroll

**Affiliations:** Medical College of Wisconsin, Milwaukee, Wisconsin, United States; Georgetown University, Washington, District of Columbia, United States; Georgetown University, Washington, District of Columbia, United States; Duke University, Durham, North Carolina, United States; University of South Florida, Tampa, Florida, United States; Memorial Sloan Kettering Cancer Center, New York, New York, United States; Georgetown University, Washington, District of Columbia, United States; University of California, Los Angeles, Los Angeles, California, United States

## Abstract

Cancer and its treatments increase risk for age-related disease, and biological aging may be a key mechanism; however, no research to date has examined epigenetic markers of aging in long-term breast cancer survivors. We used data from a national, prospective cohort to test whether older breast cancer survivors had accelerated epigenetic aging compared to non-cancer controls. Non-metastatic breast cancer survivors ages 62–84 years who received chemotherapy with or without hormonal treatment (n=29) or hormonal treatment alone (n=51), and controls frequency-matched on age, race, education, and time between blood draws (n=101), provided two blood samples between 24- and 60-months post-diagnosis (time between samples average=1.8 years). DNA methylation profiling (Illumina Infinium EPIC array) derived epigenetic aging measures: extrinsic, intrinsic, phenotypic, Grim, and Dunedin Pace of Aging (PoAm). Mixed-effects models tested effects of treatment group and change over time on epigenetic aging, adjusting for chronologic age and comorbidities. Survivors who received chemotherapy +/- hormonal treatment had a biological age 1.9–2.6 years older than controls based on extrinsic, intrinsic, and Grim estimates (p=.045, .045, and .001, respectively). Survivors who received hormonal treatment alone had an extrinsic biological age 1.6 years older than controls (p=.032) and a faster Dunedin PoAm (p=.040). Survivors who received chemotherapy +/- hormonal treatment had a trend for accelerated extrinsic aging over time compared to controls (p=.087). Older breast cancer survivors, especially those receiving chemotherapy, showed an accelerated epigenetic aging profile compared to matched women without cancer. Future research is needed to examine associations with age-related survivorship outcomes.

